# Cardiac troponin I in healthy Norwegian Forest Cat, Birman and domestic shorthair cats, and in cats with hypertrophic cardiomyopathy

**DOI:** 10.1177/1098612X221117115

**Published:** 2022-09-08

**Authors:** Sofia Hanås, Anders Larsson, Jesper Rydén, Inger Lilliehöök, Jens Häggström, Anna Tidholm, Katja Höglund, Ingrid Ljungvall, Bodil S Holst

**Affiliations:** 1 Department of Clinical Sciences, Swedish University of Agricultural Sciences, Uppsala, Sweden; 2 Evidensia Specialist Animal Hospital Strömsholm, Strömsholm, Sweden; 3 Department of Medical Sciences, Clinical Chemistry, Uppsala University, Uppsala, Sweden; 4 Department of Energy and Technology, Swedish University of Agricultural Sciences, Uppsala, Sweden; 5 Anicura Albano Animal Hospital, Stockholm, Sweden; 6 Department of Anatomy, Physiology and Biochemistry, Swedish University of Agricultural Sciences, Uppsala, Sweden

**Keywords:** hs-cTnI, breed, biomarker, feline, heart

## Abstract

**Objectives:**

The aims of this study were to assess the potential associations between the serum cardiac troponin I (cTnI) concentration in healthy cats and feline characteristics, systolic blood pressure, heart rate (HR), echocardiographic measurements and storage time; and to compare cTnI concentrations in healthy cats with concentrations in cats with hypertrophic cardiomyopathy (HCM), with or without left atrial enlargement (LAE) and in cats with HCM, to assess potential associations between cTnI concentration and echocardiographic variables.

**Methods:**

Cardiac TnI was analysed using an Abbott ARCHITECT ci16200 analyser in serum from prospectively included healthy Norwegian Forest Cat (NF; n = 33), Birman (n = 33) and domestic shorthair (DSH; n = 30) cats, and from 39 cats with HCM, with or without LAE.

**Results:**

In healthy cats, higher cTnI concentrations were found in Birman cats than in NF cats (*P* = 0.014) and in neutered male cats than in intact females (*P* = 0.032). Cardiac TnI was positively associated with HR (*P* <0.0001). In cats with HCM, cTnI concentration was positively associated with left ventricular wall thickness and with left atrial-to-aortic root ratio (all *P* ⩽0.010). Cats with HCM had higher cTnI concentrations than healthy cats, and cTnI concentrations were higher in cats with HCM and LAE than in those with HCM without LAE (all *P* = 0.0003).

**Conclusions and relevance:**

Breed and sex may affect serum cTnI concentrations in healthy cats. The cTnI concentration increased with increasing severity of HCM.

## Introduction

Cardiac troponin I (cTnI) is a sensitive biomarker for myocardial injury, and high-sensitivity cTnI (hs-cTnI) assays allow detection of low concentrations of cTnI in healthy cats,^1–4^ dogs^1,5^ and humans.^6^ An hs-cTnI assay enables the detection of cTnI concentrations in >50% of healthy humans, with a coefficient of variation (CV) of ⩽10% for the 99th percentile.^6^ In humans, different cut-off values are used for the available hs-cTnI assays because of varying capture and detection antibodies and lack of standardisation.^7,8^ Only one hs-cTnI assay has been validated in cats.^1^ In humans, male sex is positively associated with cTnI concentration, and sex-specific reference intervals (RIs) are used.^9–11^ Breed differences have been found in healthy dogs,^12,13^ and a previous report in cats with hypertrophic cardiomyopathy (HCM) indicated a breed effect.^14^ In healthy cats, cTnI concentrations have neither been associated with sex nor with breed or body condition score (BCS).^2,3,14^ Results in cats regarding associations between cTnI concentrations and age and body weight (BW) are conflicting.^2,3,15^

HCM is a common cardiac disease in cats, with a reported prevalence rate of approximately 15%.^16,17^ The disease is characterised by left ventricular hypertrophy in the absence of other explanations for wall thickening,^18^ and may lead to congestive heart failure (CHF), arterial thromboembolism (ATE), arrhythmia or sudden death.^19–21^ Using conventional cTnI assays, significantly higher concentrations have been detected in cats with HCM than in healthy cats,^22,23^ in which cTnI concentrations have been reported to be commonly below the detection limit.^23,24^ In cats with HCM, positive associations between cTnI concentrations and hypertrophy of the left ventricle (LV), and with left atrial enlargement (LAE), have been shown.^2–4,23^ Although cTnI is specific for the myocardium, increased cTnI concentrations are not specific for primary cardiac disease but have also been shown in cats with hyperthyroidism,^25^ hypertension,^26^ renal disease^27^ and critical illness.^28^

The aims of this study were to assess potential associations between serum concentration of cTnI in healthy cats and feline characteristics (breed, sex, age, BW and BCS), systolic blood pressure (SBP), heart rate (HR) at auscultation, echocardiographic measurements and storage time. A further aim was to compare cTnI concentrations in healthy cats with concentrations in cats with HCM, with and without LAE, and in cats with HCM, to assess potential associations between serum cTnI concentration in cats with HCM and echocardiographic variables. We hypothesised that the serum concentration of cTnI in healthy cats may be influenced by feline characteristics, and that cTnI concentrations may differ between healthy cats and cats with HCM, with or without LAE.

## Materials and methods

This prospective observational study was approved by the Uppsala Animal Experiment Ethics Board, Sweden (C137/13). Client-owned cats were recruited through information distributed online, at seminars or at recruiting clinics. Cats were examined at the Evidensia Animal Clinic in Västerås, Sweden, between September 2014 and June 2017. Clinically healthy cats and cats with murmurs or previously diagnosed HCM were examined for possible inclusion. Informed written consent was obtained from the owner of each cat. The population has, in part, been described previously.^29,30^

### Inclusion criteria

Apparently healthy Norwegian Forest Cat (NF), Birman and domestic shorthair (DSH) cats aged 1–14 years, with normal echocardiograms, were included, as were cats of any breed with preclinical HCM or clinical HCM stabilised following CHF therapy. A diagnosis of HCM was based on characteristic findings on an echocardiogram, as outlined below.

### Exclusion criteria

Cats with mean SBP >160 mmHg, or increased serum concentrations of total thyroxine (TT4), creatinine or alanine aminotransferase were excluded. Cats with decompensated CHF, ATE, congenital cardiac disease, other acquired cardiovascular disorders, equivocal findings concerning the presence of LV hypertrophy, or significant organ-related or systemic diseases other than HCM were excluded. All cats receiving medical treatment other than standard CHF treatment, standard antithrombotic treatment or medroxyprogesterone acetate were excluded.

### Physical examination and blood pressure measurements

All examinations were performed according to a standardised protocol in a quiet examination room by an experienced veterinarian (SH). SBP was measured indirectly using a high-definition oscillometric device (VET Memodiagnostic HDO monitor; S+B medVET),^31^ followed by a general physical examination, including assessment of BCS on a nine-point scale,^32^ echocardiography and blood sampling for haematology, biochemistry profiles, and TT4 and fructosamine levels, as previously described.^29^

### Echocardiography

Echocardiographic examination was performed using an ultrasound unit (IE33; Philips Ultrasound) equipped with a 4–12 MHz phased-array probe, and with continuous electrocardiogram monitoring.^33^ The left atrial-to-aortic root diameter ratio (LA:Ao) was measured from the right two-dimensional short-axis view.^34^ Left atrial enlargement was defined as LA:Ao ⩾1.5.^35^ End-diastolic and systolic LV dimensions (interventricular septum in diastole [IVSd], LV internal diameter in diastole [LVIDd], LV free wall in diastole [LVFWd] and LV internal diameter in systole [LVIDs]) were measured from M-mode and two-dimensional images, and fractional shortening (FS) was calculated.^33,36^

A diagnosis of HCM was made when subjective impression of hypertrophy (diffuse or regional) with a non-dilated LV chamber was supported by increased M-mode and two-dimensional diastolic LV wall dimensions of the IVSd, LVFWd or both, as previously described.^29^ Expected BW-dependent values for the IVSd, LVIDd and LVFWd, as well as percentage deviations from these expected values, were calculated for both healthy cats and cats with HCM according to previously generated formulas for cats.^35^ The calculated deviations and LA:Ao were used to classify cats into three groups – healthy controls, HCM without LAE and HCM with LAE – as described previously.^29^

### Analytical performance of hs-cTnI

The validation study was set up in accordance with the Clinical and Laboratory Standards Institute document EP15-A3, evaluating precision using a one-way ANOVA.^37^ In the imprecision study, two concentrations of pooled feline serum (cTnI concentrations 22.8 ng/l and 312.7 ng/l, respectively) and two human control samples (Liquicheck Cardiac Marker LT1 and Liquicheck Cardiac Marker Cardio 2 [Bio-Rad Laboratories]) were used. Each sample was analysed three times daily for 5 days to determine within-run (CV_R_), between-run (CV_B_) and within-laboratory (CV_WL_) repeatability. Limit of blank (LOB) was evaluated using 20 replicates of physiological saline solution. Linearity under dilution was performed in duplicate through the dilution of two concentrations of pooled feline serum (45 ng/l and 173 ng/l, respectively) with physiological saline solution as serial dilution and the addition of an extra sample consisting of 75% feline serum and 25% physiological saline solution. Recovery ([observed/expected concentration] × 100) was calculated. Stability was determined by keeping fresh feline serum samples with initial cTnI concentrations of 1070, 1040, 761 and 91 ng/l, respectively, at 20°C in the dark for 0, 3, 5 and 7 days, respectively, and then stored at −80°C until thawed for batched analysis. The effect of freezing and thawing was determined by assaying aliquots of fresh feline serum samples subjected to three cycles of freezing at −80°C for 24 h and thawing to room temperature for 30 mins.

### Analysis of cTnI in cats

Whole blood was centrifuged and the serum stored at −80°C within 60 mins of collection. Samples were transported from the clinic to the laboratory at −80°C using a portable freezer. Serum for cTnI was batch-analysed in duplicate at an accredited laboratory at the Department of Clinical Chemistry and Pharmacology, Uppsala University Hospital, using a two-step, double-monoclonal chemiluminescent microparticle immunoassay for the detection of cTnI concentration (Abbott ARCHITECT ci16200 analyser; Abbott Laboratories). The reported assay interval for cTnI concentration was 2–50,000 ng/l. Concentrations <2 ng/l were assigned a value of 2 ng/l in the calculations.

### Statistical analysis

Statistical analyses were performed using Rstudio^38^ and JMP (version 12.2.0; SAS Institute). A *P* value <0.05 was considered to be statistically significant. Descriptive statistics for continuous variables (age, BW, SBP, HR, basic echocardiographic measurements and basic laboratory variables) were presented as mean ± SD. Within each of these continuous variables, multiple comparisons were performed between breeds for healthy cats, and between healthy cats and cats with HCM, using one-way ANOVA followed by Tukey’s honestly significant difference (HSD) test.

Data on cTnI concentrations were presented as median and interquartile range (IQR). Potential associations between cTnI concentration in healthy cats, as well as in cats with HCM and the variables breed, sex, BCS, age, BW, SBP, HR, echocardiographic measurements IVSd_inc%_, LVIDd_inc%_, LVFWd_inc%_, FS% and LA/Ao, and the duration of storage at −80°C of serum samples, were analysed using univariable linear regression for continuous variables and one-way ANOVA for categorical variables. BCS was divided into normal (BCS 4–5) and overweight (BCS 6–7), and sex into four classes (male, female, neutered male and neutered female). Variables with a *P* value <0.2 were included in a linear multiple regression analysis and, as cTnI is a marker of myocardial injury, a variable for the LV wall – LVPWd_inc%_ – was also included in the model for healthy cats. A model selection was performed, excluding covariate echocardiographic variables for the LV, using the echocardiographic variable with the lowest *P* value in the model. The variable with the highest *P* value was then removed until all remaining variables had a *P* value <0.05. The model with the lowest Akaike information criterion was chosen. All variables were assessed as main effects. The interactions breed × BW and breed × sex were evaluated but were not significant and thus not included in the model. All multiple regression analyses were performed after logarithmic transformation of cTnI concentrations, to have normally distributed residuals. For multiple comparisons between sex and breed categories, respectively, Tukey’s HSD test was used as implemented by the routine general linear hypotheses in the R package ‘multcomp’.

Comparisons of cTnI concentrations between the healthy, HCM without LAE and HCM with LAE groups were done using the Kruskal–Wallis test, followed by post-hoc Wilcoxon tests for each pair.

## Results

### Validation of the hs-cTnI immunoassay

The CV_R_, CV_B_ and CV_WL_ were 4.0–8.6% for the feline samples and 5.1–8.4% for the human control samples. Assay results were adequately linear after dilution and the recovery was 64–116%. The LOB was 0.25 ± 0.12 ng/l. After 3 days of storage at room temperature, the concentrations of cTnI were still within 10% in comparison with the initial values (see Supplementary Appendix 1 and Supplementary Figure 1 in the supplementary material).

### Study population

#### Healthy cats

**Figure 1:**
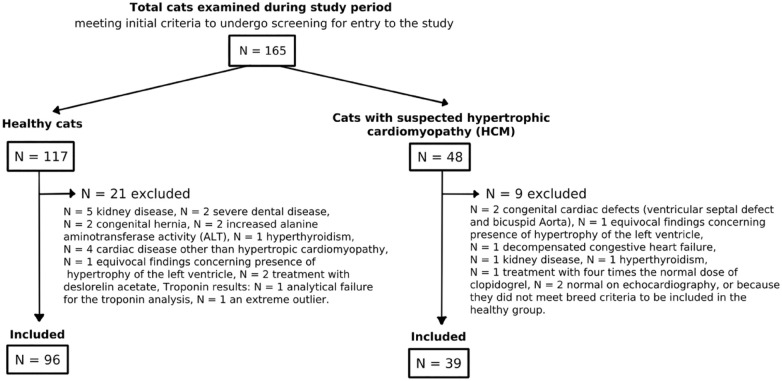
Flow chart of the study, including the reasons for the exclusion of 30 cats

The 96 healthy cats comprised Birman, NF and DSH cats. The characteristics of the healthy cats are presented in Table 1 and their basic echocardiographic data in Supplementary Table 1; excluded cats are detailed in Figure 1.

**Table 1:** Feline characteristics, heart rate (HR) at auscultation and systolic blood pressure (SBP) in 96 healthy cats

Group	Birman (n = 33)	DSH (n = 30)	NF (n = 33)
Sex			
Female	12	0	10
Male	5	0	3
Female neutered	9	14	12
Male neutered	7	16	8
Age (years)	4.8 ± 4.0^a^	7.0 ± 4.4^a^	5.0 ± 3.0^a^
Body weight (kg)	3.6 ± 0.7^a^	4.7 ± 1.1^b^	5.4 ± 1.6^b^
Normal*	21	12	14
Overweight^†^	11^‡^	18	19
HR (bpm)	159 ± 20^a^	151 ± 27^a^	165 ± 27^a^
SBP (mmHg)	125 ± 11^a^	140 ± 10^b^	134 ± 13^b^

Data are provided mean ± SD for continuous variables. Within each row, values with different superscripts differ significantly between groups. Statistical significance was set at *P* <0.05. Multiple comparisons within each independent variable were corrected using Tukey’s method

* Body condition score (BCS) 4–5/9

† BCS 6–7/9

‡ One missing value

DSH = domestic shorthair; NF = Norwegian Forest Cat; LAE = left atrial enlargement; HR = heart rate at auscultation; bpm = beats/min; SBP = systolic blood pressure

#### Cats with HCM

The 39 cats with HCM comprised 18 DSH and cats from 10 different breeds (five Persian, four NF, two Bengal, two Maine Coon, two Ragdoll and one each of the following breeds: British Shorthair, Cornish Rex, Devon Rex, Exotic Shorthair, Siberian and Sphynx). Of 32 cats with HCM without LAE, one female was treated with medroxyprogesterone acetate, and three were treated with enalapril. Of the seven cats with HCM and LAE, three cats with stabilised CHF were treated with furosemide and enalapril, one of which also received clopidogrel. The characteristics of the included cats with HCM are presented in Table 2 and their basic echocardiographic data in Supplementary Table 2; excluded cats are detailed in Figure 1.

**Table 2:** Feline characteristics, heart rate (HR) at auscultation, systolic blood pressure (SBP) and selected echocardiographic data in 96 healthy cats and 39 cats with hypertrophic cardiomyopathy (HCM)

Group	Healthy (n = 96)	HCM without LAE (n = 32)	HCM with LAE (n = 7)
Sex			
Female	22	3	1
Male	8	4	0
Female neutered	35	9	0
Male neutered	31	16	6
Age (years)	5.6 ± 3.9^a^	6.1 ± 3.4^a^	6.3 ± 2.7^a^
Weight (kg)	4.6 ± 1.4^a^	5.1 ± 1.2^a^	4.8 ± 1.1^a^
Normal*	47	6	5
Overweight^†^	48^‡^	22^§^	2
HR (bpm)	158 ± 25^a^	167 ± 26^a^	169 ± 31^a^
SBP (mmHg)	133 ± 13^a^	138 ± 12^a^	128 ± 11^a^
LA:Ao	1.1 ± 0.1^a,¶^	1.1 ± 0.1^a,¶^	1.6 ± 0.1^b,¶^
LVFWd (mm)	3.8 ± 0.5^a,¶^	5.4 ± 1.3^b,¶^	7.6 ± 0.5^c,¶^
LVFWd_inc%_	−0.9 ± 9.5^a,¶^	39.6 ± 33.2^b,¶^	99.8 ± 16.7^c,¶^

Mean ± SD is provided for continuous variables. Within each row, values with different superscripts differ significantly between groups. Multiple comparisons within each independent variable were corrected using Tukey’s method. The level of statistical significance was set at *P* <0.05

* Body condition score (BCS) 4–5/9

† Overweight = BCS 6–7/9

‡ One missing value

§ Four missing values

¶ Expected differences due to echocardiographic classification of cats into three groups: healthy controls, HCM without LAE, and HCM with LAE

HR = heart rate at auscultation; LAE = left atrial enlargement; bpm = beats/min; SBP = systolic blood pressure; LA:Ao = left atrial-to-aortic root diameter ratio; LVFWd = left ventricular free wall in diastole; LVFWd_inc%_ = percentage increase left ventricular free wall in diastole

### Association between cTnI and feline characteristics, and clinical and echocardiographic variables in healthy cats

cTnI was >2 ng/l in 89/96 samples (93%). cTnI was significantly associated with breed and sex, and positively associated with HR (Tables 3 and 4) but not with age, SBP within the normal range or LVFWd_inc%_. Higher cTnI concentrations were found in Birman cats than in NF cats (*P* = 0.014), and in neutered males than in intact females (*P* = 0.032; Tables 3 and 4). The final multiple regression model included breed, sex, HR and LVFWd_inc%_ (Table 4).

**Table 3:** Serum concentration of cardiac troponin I (cTnI) in 96 healthy cats

Group	n	Median (IQR) cTnI (ng/l)	Range (ng/l)
Breed			
NF	33	4.0 (2.6–7.7)^a^	<2.0–42.0
Birman	33	7.6 (3.3–14.4)^b^	<2.0–49.0
DSH	30	7.7 (3.0–16.1)^a,b^	<2.0–156.0
Sex			
Intact female	22	3.7 (2.1–7.5)^a^	<2.0–20.0
Intact male	8	7.8 (5.5–10.5)^a,b^	<2.0–24.0
Female neutered	35	6.3 (3.3–11.5)^a,b^	<2.0–156.0
Male neutered	31	7.5 (3.0–16.0)^b^	<2.0–61.5
All cats	96	5.7 (2.8–11.0)	<2.0–156.0

Different superscripts within a column denote differences in cTnI concentrations between breed and sex, respectively. The level of statistical significance level was set at *P* <0.05. Multiple comparisons within breed and sex were adjusted using Tukey’s method

IQR = interquartile range; NF = Norwegian Forest Cat; DSH = domestic shorthair

**Table 4:** Final model in the multiple regression analysis of association between the effects of feline characteristics and echocardiographic variables and heart rate (HR) at auscultation on the serum concentration of cardiac troponin I (cTnI) in 96 healthy cats

Variable	Estimate	*P* value	Adjusted *P* value	95% CI
NF	Baseline breed			
Birman	0.589	0.005	0.014	0.133–1.045
DSH	0.521	0.023	0.058	0.080–0.961
Female sex	Baseline sex			
Male sex	0.417	0.22	0.599	−0.245 to 1.079
Female neutered	0.483	0.045	0.178	0.017–0.948
Male neutered	0.708	0.0067	0.032	0.209–1.208
HR (bpm)	0.017	<0.0001		0.010–0.024
LVFWd_inc%_	0.012	0.19		−0.006 to 0.030

The estimate column gives the estimated regression coefficients in the fitted regression model. cTnI is a marker of myocardial injury, and thus a variable for the left ventricular wall (LVPWd_inc%_) was included in the model. The level of statistical significance was set at *P* <0.05. Multiple comparisons within breed and sex, respectively, were adjusted using Tukey’s method. The final model had an r^2^ of 0.23 and an overall *P* value of <0.0001

CI = confidence interval; NF = Norwegian Forest Cat; DSH = domestic shorthair; HR = heart rate at auscultation; bpm = beats/min; LVFWd_inc%_ = percentage increase left ventricular free wall in diastole (percentage)

### Association between cTnI and HCM

The median cTnI serum concentration in cats with HCM was 37 ng/l (IQR 17.5–81.5), and was higher in cats with HCM with LAE than in cats with HCM without LAE or in healthy cats (*P* <0.0001; Table 5). All three groups differed (all *P* = 0.0003; Table 5). For the analysis of the 39 cats with HCM, the final model included the variables LWFWd_inc%_ and LA/Ao, both positively associated with cTnI (Table 6).

**Table 5:** Serum concentration of cardiac troponin I (cTnI) vs echocardiographic classification in the study population of 135 cats

Group	n	Median (IQR) cTnI (ng/l)	Range (ng/l)
Healthy controls	96	5.7 (2.8–11.0)^a^	<2.0–156.0
HCM without LAE	32	29.3 (13.3–46.5)^b^	3.1–318.0
HCM with LAE	7	296 (92.0–642.0)^c^	56.0–1880.0

Different superscripts within a column denote differences in cTnI concentrations between the groups (healthy controls, HCM without LAE and HCM with LAE). The level of statistical significance was set at *P* <0.05. For multiple comparisons, Kruskal–Wallis test was used, followed by post-hoc Wilcoxon tests for each pair

HCM = hypertrophic cardiomyopathy; LAE = left atrial enlargement; IQR = interquartile range

**Table 6:** Final model in the multiple regression analysis of association between the effects of echocardiographic variables on the serum concentration of cardiac troponin I in 39 cats with hypertrophic cardiomyopathy

Variable	Estimate	*P* value	95% CI
LVFWd_inc%_	0.016	0.004	0.006–0.026
LA:Ao	2.41	0.010	0.66–4.15

The estimate column gives the estimated regression coefficients in the fitted regression model. The level of statistical significance was set at *P* <0.05. The final model had an adjusted r^2^ of 0.46 and an overall *P* value of <0.0001

CI = confidence interval; LA:Ao = left atrial-to-aortic root diameter ratio; LVFWd_inc%_ = percentage increase of the left ventricular free wall in diastole

## Discussion

In the present study, the hs-cTnI assay was precise and accurate for use in cats. The main findings were a significant association between serum cTnI concentrations and breed, sex and HR in healthy cats. There was no significant association between cTnI concentration and age, or with SBP within the normal range. There were significant differences in serum cTnI concentration between healthy cats, cats with HCM without LAE and cats with HCM with LAE, which is in accordance with previous studies in cats.^2,3^

In healthy cats, higher cTnI concentrations were found in Birman cats than in NF cats. Interbreed variations have previously been reported in dogs, with higher cTnI concentrations in Greyhound and Boxer dogs than in other breeds,^12,13^ but not in healthy cats.^2,3,14^ In cats with HCM, a previous study indicated a breed effect with higher cTnI concentrations in British Shorthair cats than in Maine Coons.^14^ However, echocardiographic variables indicating the severity of HCM were not included in the statistical analysis in that study,^14^ thus the British Shorthair cats may simply have had more severe HCM at inclusion than the Maine Coons. An effect of sex was also found in healthy cats, with higher cTnI concentrations in male neutered cats than in intact female cats. In humans, women have been described as having lower cTnI concentrations than men.^9,39^ In the present study, intact female cats had lower concentrations of cTnI than intact males. The observed difference was not statistically significant, but this may have been a type II statistical error due to the small number of intact cats included in the study. Healthy cats had comparatively lower serum cTnI concentrations, and the breed and sex associations were probably detected owing to the more sensitive cTnI assay used in the present study, as compared with previously used assays in cats.^2,3,14^ However, despite being statistically significant, differences in cTnI concentrations among breed and sex were much smaller than between healthy cats and cats with HCM, and likely not of relevance in a clinical situation. Further studies would be needed to investigate if breed- and sex-specific RIs are beneficial.

The lack of association between age and cTnI concentration is in contrast to reports on healthy people,^40,41^ healthy dogs^42^ and dogs with mild myxomatous mitral valve disease,^43^ where cTnI has been positively associated with age. In cats, both a positive association^15^ and no association^2,3^ with age have been previously described. In the present study, the mean age of the healthy cats was 5.6 years, with few older cats, which possibly contributed to the lack of association.

In the present study, a positive association between cTnI concentration and HR was found in healthy cats. Prolonged exercise or stress increases HR and blood pressure.^44–46^ In humans^47^ and in horses,^48–50^ post-exercise-induced cTnI elevations have been reported after normal physical activity, with a peak at 3–6 h post-exercise and normalisation within 24 h.^49,51^ The increase of circulating cTnI after exercise has been suggested to arise from a proportion of cTnI that is free in the cytosol,^52^ and thus not a sign of cardiac damage. In the present study, blood sampling was performed a couple of hours after leaving home, and situational stress might be an explanation for higher HR.

cTnI concentration differed significantly between healthy cats, cats with HCM without LAE and cats with HCM with LAE. Previous investigations in humans,^53^ cats^4,54^ and dogs^43^ suggest that cTnI correlates with the severity of HCM and myxomatous mitral valve disease. Echocardiography is used for the diagnosis of LV hypertrophy and the HCM phenotype in cats.^18,55–57^ Diagnosis is based on a subjective impression of LV hypertrophy supported by measurement of maximal end-diastolic wall thicknesses via two-dimensional or M-mode echocardiography.^58–60^ Fixed diagnostic cutoffs defining normal diastolic LV wall thickness have been used.^55,60,61^ Several studies have recommended allometric scaling and BW-based 95% prediction intervals, in particular in small and large cats.^35,62,63^ In the present study, BW-dependent echocardiographic values, as well as percentage deviations from these expected values, were calculated and used. Evaluations of the cTnI concentration for assessing HCM in cats categorised using echocardiographic measurements adjusted for BW have, to our knowledge, not been published previously. Cats with HCM and LAE have a more severe disease than cats with HCM without LAE.^20,64–66^ In a study with a median follow-up of 3.1 years, cats with HCM and LAE were four times more likely to experience a cardiac event than cats with a normal atrial size.^67^ In accordance with previous studies in cats, concentrations of cTnI in cats with HCM were positively associated with LVFWd_inc%_ and LA:Ao,^2,3^ possibly caused by myocardial ischaemia due to myocyte death or by an imbalance between hypertrophy of the myocardium and insufficient coronary arterial supply.^68,69^ Although cTnI is heart-specific, increases are not specific for conditions affecting the heart, and increased cTnI concentrations have been described in cats with hyperthyroidism,^25^ hypertension,^26^ renal disease^27^ and critical illness.^28^

Concentrations of cTnI differ substantially between different hs-cTnI assays,^7^ and concentrations obtained with different hs-cTnI assays are therefore difficult to compare. In the present study, cTnI was detectable in 93% of healthy cats, which is higher than previously reported in cats evaluated using another hs-cTnI assay.^2^ The CVs in our study were comparable to those previously reported for cTnI assays in humans,^70,71^ dogs^1,72^ and cats.^1^ The concentration of cTnI in samples stored at 20°C for 3 days changed by less than 10%, possibly enabling the transport of fresh feline serum samples to a central laboratory. However, stability was only studied for cTnI concentrations above 90 ng/l, and studies of concentrations closer to the clinical cutoff would be valuable. In the present study, cTnI analyses for the healthy cats and the cats with HCM were performed after up to 5 years of serum storage at −80°C, but storage time was not associated with cTnI concentration in the statistical analysis.^71^

The design of our study, with different criteria for selection of the healthy controls and the cats with HCM (ie, three specific breeds were included as healthy controls and any breed was permitted in the HCM cat group), is a limitation. The different breed distributions may have affected the results because breed influences the cTnI concentration. Only healthy cats and cats with HCM, but no other known diseases, were included in the study. Cats with equivocal echocardiographic findings and measurements, or other comorbidities were excluded, which introduces the spectrum effect for cTnI concentration.^73^ In a mixed cat population, including cats with comorbidities, the rate of elevated cTnI results would increase and the accuracy for the test for discriminating healthy cats from cats with HCM is likely to decrease. The present study was intended as an exploratory study, and further research in a mixed cat population is warranted for a discriminatory analysis. Body-weight dependent echocardiographic values were used for both healthy cats and cats with HCM, which affected the ability to compare the results to those of previous studies that have used a fixed diagnostic cutoff for HCM. A fixed cutoff may have increased the inclusion of mild HCM in the healthy control group, especially if the cats were small, and extremely large cats may have been falsely included as mild HCM cats.

## Conclusions

Analytical performance of the hs-cTnI analysis allows its clinical use in cats. There was an effect of both breed and sex on the serum concentration of cTnI. In cats with HCM, cTnI concentrations increased with increasing LV wall thickness, and cats with LAE had higher cTnI concentrations than cats with HCM without LAE and than healthy cats.

## Supplemental Material

Supplement 1 Stability study Click here for additional data file. The concentration of cTnI decreased in serum samples stored at 20°C. After three days, the mean decrease was 4% in comparison with the initial value. Mean decrease was 14% after 5 days and 20% after 7 days (Supplement Figure 1). The cTnI concentration changed from -1 to 5% after three freeze-thaw cycles.

Supplement Table 1 Click here for additional data file. Auscultation, basic echocardiographic and laboratory variables in 96 healthy Birman, Domestic Shorthair (DSH), and Norwegian Forest (NF) cats

Supplement Table 2 Click here for additional data file. Auscultation, basic echocardiographic and laboratory variables in 96 healthy cats and 39 cats with hypertrophic cardiomyopathy (HCM)

Supplement Figure 1 Click here for additional data file. The stability of cardiac troponin I (cTnI) concentration in serum samples from three cats with hypertrophic cardiomyopathy (HCM) after storage in the dark at 20°C
From cat one, two samples were studied (Cat 1a and Cat 1b).
