# Supplementary material for: Cardiac troponin I in healthy Norwegian Forest Cat, Birman and domestic shorthair cats, and in cats with hypertrophic cardiomyopathy
Source: J Feline Med Surg. 2022 Sep 8;24(10):e370–9. doi: 10.1177/1098612X221117115 (PMC9511503; doi:10.1177/1098612X221117115)
Supplement: Supplement 1 Stability study [file sj-docx-1-jfm-10.1177_1098612X221117115.docx]

**Supplement 1 Stability study**

The concentration of cTnI decreased in serum samples stored at 20°C. After three days, the mean decrease was 4% in comparison with the initial value. Mean decrease was 14% after 5 days and 20% after 7 days (Supplement Figure 1). The cTnI concentration changed from -1 to 5% after three freeze-thaw cycles.

**Supplement Figure 1** The stability of cardiac troponin I (cTnI) concentration in serum samples from three cats with hypertrophic cardiomyopathy (HCM) after storage in the dark at 20°C

From cat one, two samples were studied (Cat 1a and Cat 1b).

[Insert Supplement Figure 1]
