# Supplementary material for: Cardiac troponin I in healthy Norwegian Forest Cat, Birman and domestic shorthair cats, and in cats with hypertrophic cardiomyopathy
Source: J Feline Med Surg. 2022 Sep 8;24(10):e370–9. doi: 10.1177/1098612X221117115 (PMC9511503; doi:10.1177/1098612X221117115)
Supplement: Supplement Table 1 [file sj-docx-3-jfm-10.1177_1098612X221117115.docx]

**Supplement Table 1** Auscultation, basic echocardiographic and laboratory variables in 96 healthy Birman, Domestic Shorthair (DSH), and Norwegian Forest (NF) cats

| **Group** | **Birman** | **DSH** | **NF** |
| --- | --- | --- | --- |
| Murmur (yes/no) | 2/31 | 0/30 | 1/32 |
| *Basic echocardiographic data* | | | |
| LA/Ao | 1.1 ± 0.1^a^ | 1.1 ± 0.1^a^ | 1.1 ± 0.1^a^ |
| IVSd (mm) | 3.6 ± 0.4^a^ | 3.9 ± 0.4^b^ | 4.0 ± 0.4^b^ |
| IVSd_inc%_ | -1.5 ± 9.1^a^ | 0.4 ± 8.8^a^ | -0.6 ± 9.4^a^ |
| LVIDd (mm) | 15.1 ± 1.6^a^ | 16.5 ± 1.9^b^ | 17.1 ± 2.3^b^ |
| LVIDd_inc%_ | 1.5 ± 8.8^a^ | 3.1 ± 11.9^a^ | 3.3 ± 10.0^a^ |
| LVFWd (mm) | 3.5 ± 0.4^a^ | 3.8 ± 0.5^b^ | 3.9 ± 0.4^b^ |
| LVFWd_inc%_ | -2.2 ± 9.5^a^ | 0,1 ± 10.4^a^ | -0.5 ± 8.8^a^ |
| FS (%) | 49 ± 7^a^ | 52 ± 7^a^ | 50 ± 7^a^ |

LA/Ao = left atrial-to-aortic root diameter ratio; IVSd = interventricular septum in diastole; IVSd_inc_ = percentage increase interventricular septum in diastole; LVIDd = left ventricular internal diameter in diastole; LVIDd_inc_ = percentage increase left ventricular internal diameter in diastole; LVFWd = left ventricular free wall in diastole; LVFWd_inc_ = percentage increase left ventricular free wall in diastole; FS = fractional shortening. The mean ± SD is shown for continuous variables. Significance level was set at *P* < 0.05. Multiple comparisons within each independent variable were corrected using Tukey’s method. Within each row, values with different superscripts differ significantly between breeds.
