# Supplementary material for: Cardiac troponin I in healthy Norwegian Forest Cat, Birman and domestic shorthair cats, and in cats with hypertrophic cardiomyopathy
Source: J Feline Med Surg. 2022 Sep 8;24(10):e370–9. doi: 10.1177/1098612X221117115 (PMC9511503; doi:10.1177/1098612X221117115)
Supplement: Supplement Table 2 [file sj-docx-4-jfm-10.1177_1098612X221117115.docx]

**Supplement Table 2** Auscultation, basic echocardiographic and laboratory variables in 96 healthy cats and 39 cats with hypertrophic cardiomyopathy (HCM)

| **Group** | **Healthy** | **HCM without LAE** | **HCM with LAE** |
| --- | --- | --- | --- |
| Murmur (yes/no) | 3/93 | 30/2 | 7/0 |
| *Basic echocardiographic data* | | | |
| IVSd (mm) | 3.8 ± 0.4^a^ * | 5.9 ± 1.1^b^ * | 7.1 ± 1.5^c^ * |
| IVSd _inc%_ | -0.6 ± 9.0^a^ * | 51.2 ± 26.0^b^ * | 81.7 ± 31.7^c^ * |
| LVIDd (mm) | 16.2 ± 2.1^a^ | 14.9 ± 2.5^b^ | 14.8 ± 2.9^a,b^ |
| LVIDd_inc%_ | 2.6 ± 10.2^a^ | -8.4 ± 15.0^b^ | -6.9 ± 22.3^a,b^ |
| Septum 2Dd (mm) | 4.0 ± 0.5^a^ * | 6.9 ± 1.0^b^ * | 8.3 ± 1.0^c^ * |
| FS (%) | 50 ± 7^a^ | 59 ± 7^b^ | 50 ± 9^a^ |
| SAM (yes/no) | 0/96 | 24/8 | 5/2 |
| *Basic laboratory variables* | | | |
| Hematocrit (%) | 36 ± 4^a,1^ | 40 ± 21^a^ | 33 ± 3^a^ |
| Total protein (g/l) | 72 ± 6^a,1^ | 72 ± 4^a^ | 70 ± 9^a^ |
| TT4 (nmol/l) | 31 ± 7^a^ | 30 ± 7^a^ | 33 ± 8^a^ |
| Creatinine (µmol/l) | 148 ± 27^a^ | 137 ± 30^a^ | 160 ± 20^a^ |

IVSd = interventricular septum in diastole; IVSd_inc%_ = percentage increase interventricular septum in diastole; LVIDd = left ventricular internal diameter in diastole; LVIDd_inc%_ = percentage increase left ventricular internal diameter in diastole; FS = fractional shortening; Septum 2Dd, septum diameter in two dimensional mode in diastole; SAM = systolic anterior motion of the mitral valve; TT4 = total thyroxine. The mean ± SD is shown for continuous variables. Significance level was set at *P* < 0.05. . Multiple comparisons within each independent variable were corrected using Tukey’s method. Within each row, values with different superscripts differ significantly between groups. ^1^ = one missing value. *= expected differences due to echocardiographic classification cats into three groups: healthy controls, HCM without LAE, and HCM with LAE.
