# Supplementary figures and images for: Cardiac troponin I in healthy Norwegian Forest Cat, Birman and domestic shorthair cats, and in cats with hypertrophic cardiomyopathy
Source: J Feline Med Surg. 2022 Sep 8;24(10):e370–9. doi: 10.1177/1098612X221117115 (PMC9511503; doi:10.1177/1098612X221117115)

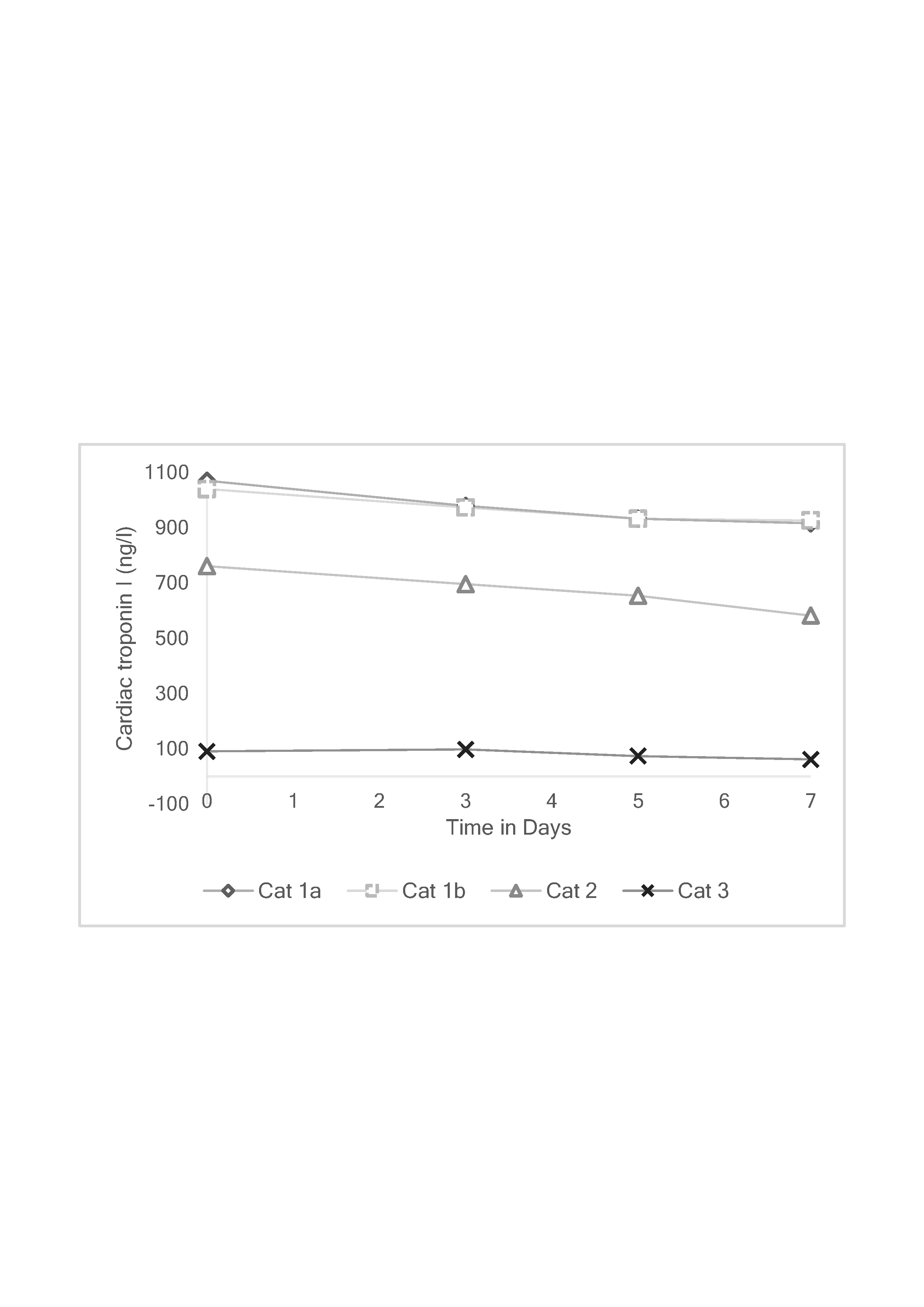

Supplement: Supplement Figure 1 [file sj-tiff-2-jfm-10.1177_1098612X221117115.tiff]
